# Anatomical variability of the anterolateral thigh flap perforators between sexes: a cadaveric study

**DOI:** 10.1007/s00238-012-0778-z

**Published:** 2012-11-08

**Authors:** Mateusz Zachara, Piotr Drozdowski, Mariusz Wysocki, Ireneusz Siewiera, Piotr Wójcicki

**Affiliations:** 1 Department of Plastic Surgery, Medical Centre, ul. Jana Pawła II 2, 57-320 Polanica-Zdrój, Poland; 2 Department of Plastic Surgery, Wrocław Medical University, Polanica-Zdrój, Poland

**Keywords:** Anterolateral thigh flap, Perforators, Oblique branch, Cadaver study, Sex

## Abstract

**Background:**

Anterolateral thigh flap (ALTF) has gain popularity as a workhorse flap in the management of simple as well as complex tissue defects. The purpose of this study was to investigate the differences in ALTF’s perforators’ location in male and female human cadavers.

**Methods:**

The study involved 30 fresh human cadavers of both sexes. A total of 60 flaps were examined. The flaps were raised as originally designed. After location of vessels, the distance from the anterior superior iliac spine (ASIS) to subsequent perforators was measured. Also, the kind of the perforator, its diameter and origin were marked. Perforators were designated according to Yu’s classification (A, B, and C). The perforators were divided into thin (<0.5 mm), medium (0.5–1 mm), and thick (>1 mm). Ratio of the ASIS–patella distance to the distance of a given perforator from the ASIS (AP rate) was calculated.

**Results:**

The mean AP rate (perforator location) was different in both sexes. Mean AP rate in men was calculated as 0.498 ± 0.117, and in women, 0.559 ± 0.114. Differences in AP rate between female and male were statistically significant (*t* = −3.144; *p* < 0.002). Mean flap thickness was 3.65 cm in women and 1.17 cm in men (*t* = −14.444; *p* < 0.00001). In men, 63 perforators originated from descending branch, and seven perforators originated from oblique branch. In women, there were 67 and one, respectively.

**Conclusions:**

In men, perforators are located closer to the ASIS in comparison to women. Clinically significant perforators (*Φ* > 0.5 mm), in majority of cases, occur in A and B positions. Thickness of the flap was higher in women. The oblique branch was more common in men.

## Introduction

Three new flaps localized on the posterior, anteromedial, and anterolateral surfaces of the thigh were described in 1984 [1]. The latter has gained popularity and was presented in the literature as “the workhorse flap” [2] in the management of simple as well as complex tissue defects [3–5]. The first description concerned a septocutaneous perforator-based flap [1]. Progress in the knowledge on topography of perforators has contributed to the success of the anterolateral thigh flap (ALTF), which was described by Wei as “an ideal flap,” one of few flaps developed for widespread applications in soft tissue reconstruction [6].

Arterial supply of the flap by either septocutaneous or musculocutaneous perforator is well documented. The lateral circumflex femoral artery (LCFA), which gives off the ascending, transverse, and descending branches, is the first branch of the deep femoral artery. The anterolateral thigh flap in majority of cases is supplied by the descending branch [7]. In its initial segment, it runs obliquely in a groove between the rectus femoris and vastus lateralis muscles, and next, it enters the latter and locates in its upper pole, terminating near the knee joint. The descending artery is accompanied by two veins [8]. The arterial vessel diameter is 2 to 2.5 mm (average 2.1 mm); veins, 1.8 to 3.3 mm (average 2.5 mm), and the length of the pedicle is 8 to 16 cm (average 12 cm) [7, 8].

The descending branch gives off perforating vessels which supply the skin on the anterolateral aspect of the thigh. The vessels are of two kinds. The septocutaneous vessels pass through the intermuscular septum between the rectus femoris and vastus lateralis muscles and supply the skin directly. They occur in 12–40 % of cases. The musculocutaneous perforators pass through the vastus lateralis muscle. They occur in 60–88 % of cases [4, 6, 9–14].

Perforators in majority of cases are localized in the central part along the line joining the anterior superior iliac spine (ASIS) and the superolateral border of the patella [15, 16]. The course, kinds, locations, and number of perforators are changeable. Also, location and course of the descending branch are irregular [17–20]. Although the descending branch is regarded to be the primary ALTF vessel, a number of recent reports have suggested a different anatomical structure [3, 21, 22]. In the study by Kimata, out of 74 patients, 16 % represented such a variability [21]. Shieh et al. observed it in as many as 34 % of the subjects [3]. This formerly unknown branch was termed “the oblique branch.” It usually runs between the transverse and descending branches. Raising the flap on this vessel spares the descending branch, which is the main artery supplying for the vastus lateralis muscle [22].

The advantages of ALTF include long pedicle, suitable vessels diameter, the possibility of thinning the flap, as well as harvesting various tissues, e.g., fascia or muscle. It is possible to raise an innervated flap, a double island flap, a flow-through flap, or a chimeric flap, combined with other flaps, e.g., fibular or iliac flap. The additional advantages of ALTF are concerned with low donor site morbidity and the fact that the procedure is performed by two teams simultaneously, which significantly shorten the operation time.

Technical limitations include unstable blood supply and, sometimes, lack of perforator with an adequate diameter. Hair coverage and diverse color of the skin may hamper the applicability in the head and neck regions [5, 6, 9, 23].

The aim of the study was as follow:

1. To describe and compare in both sexes the blood supply of the anterolateral thigh flap on human cadavers and
2. To compare the blood supply of the anterolateral thigh flap on both sides of the body in individuals of both sexes.

The study protocol was approved by the Bioethical Committee at Wrocław Medical University (no. KB12/2009).

## Material and methods

**Fig. 1:**
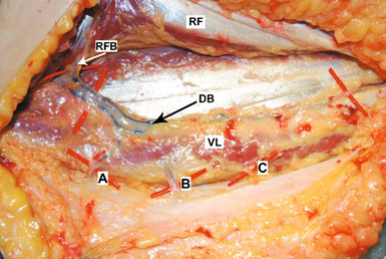
Cadaveric specimen demonstrating descending branch (*DB*); perforators *A*, *B*, and *C*; vastus lateralis (*VL*); rectus femoris (*RF*); and rectus femoris branch (*RFB*)

The study involved 30 human cadavers, 15 males and 15 females, aged from 30 to 89 years (mean age, 71). No scars were revealed on the thighs. A total of 60 flaps were examined. The distance between the anterior superior iliac spine and upper lateral border of the patella was measured prior to flap elevation. The flap was raised as originally designed; the skin was incised 2–3 cm above the longitudinal line joining ASIS and the lateral margin of the patella, down through the deep fascia to locate the perforating vessels. They were designated according to Yu et al.’s classification (Fig. 1) [17]. After preparation of the vessels, the distance from ASIS to subsequent perforators was measured at the point of their passage through the fascia. Also, the kind of perforator and its diameter were marked by means of a measure on a microvascular clamp. The perforators were divided into thin (<0.5 mm), medium (0.5–1 mm), and thick (>1 mm). In order to standardize the obtained findings (i.e., to eliminate the effect of the length of the thing in every individual), coefficients reflecting the ratio of the iliac spine–patella distance to the distance of a given perforator from the iliac spine (AP rate) were calculated.

Statistical analysis was performed using Statistica 8.0 (StatSoft, Inc.). All obtained data were analyzed using *t* test or *χ* ^2^ test. Statistical significance was set at *p* < 0.05.

## Results

**Fig. 2:**
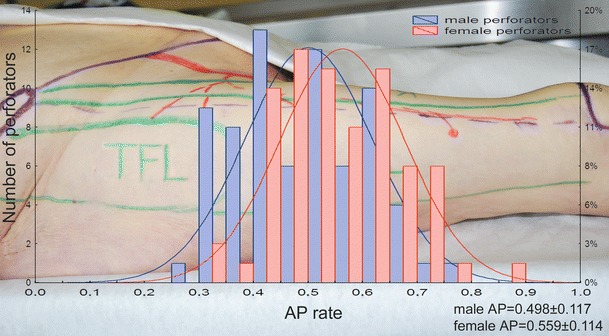
Differences between female and male thighs in distribution of AP rate

**Fig. 3:**
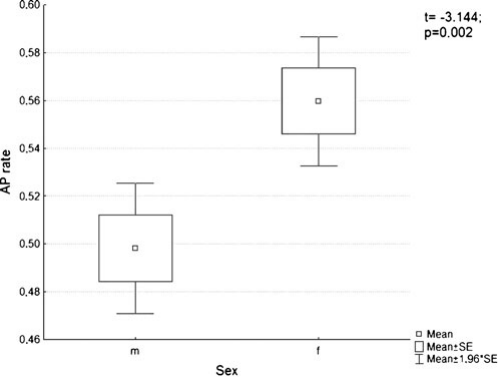
A *t* test revealed that perforators in male thigh were statistically closer to the ASIS (*t* = −3.144; *p* < 0.002)

**Fig. 4:**
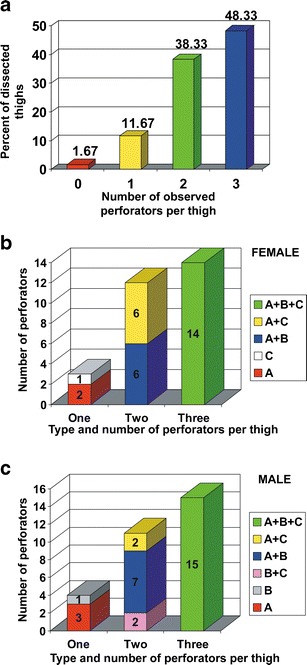
**a** Distribution of perforators’ number observed in dissected thighs. Either in female (**b**) or male (**c**) thighs, most frequently, three perforators were dissected; however, in similar number of thighs, there were two perforators identified in different configurations

**Fig. 5:**
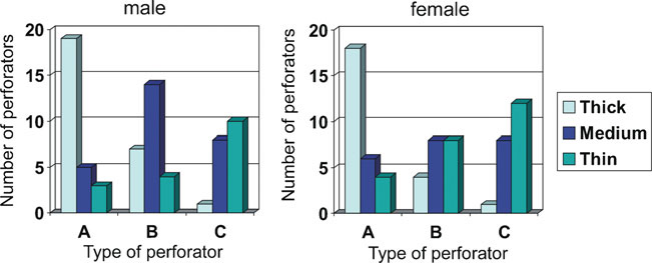
Both, in male and female thighs, clinically relevant perforators (*Φ* > 0.5 mm) significantly more often occurred as A or B perforators (male *χ* ^2^ = 11.72, *p* = 0.0006; female *χ* ^2^ = 6.65, *p* = 0.009), and in both sexes, perforator A was most often identified as thick perforator (male *χ* ^2^ = 17.19, *p* < 0.000001; female *χ* ^2^ = 18.04, *p* < 0.000001)

In men, 71 perforators were identified, 53 musculocutaneous perforators and 18 septocutaneous vessels, while in women, there were 69 perforators, 45 musculocutaneous and 24 septocutaneous ones. In total, 140 perforators were identified in both sexes, including 98 musculocutaneous (70 %) and 42 septocutaneous (30 %) kinds. In this number, there were 50 thick perforators (with a diameter >1 mm; 27 in men and 23 in women), 49 medium perforators (0.5–1 mm; 27 in men and 22 in women), and 41 thin perforators (<0.5 mm; 17 in men and 24 in women) (Table 1). Comparison of the AP rates demonstrated that location of the perforators on the anterolateral surface of the thigh differs in both sexes. Mean AP rate in men (0.498 ± 0.117) means that the vessels are located in close proximity to the ASIS. In women, they were situated further away from the ASIS (AP = 0.559 ± 0.114) (Fig. 2). Differences in AP rates between female and male were statistically significant (*t* = −3.144; *p* < 0.002) (Fig. 3). A similar statistically significant result was obtained when only clinically significant perforators (>0.5 mm in diameter) were taken into account—0.474 ± 0.1 for men and 0.537 ± 0.12 for women (*t* = −2.822; *p* < 0.005). The number of perforators differed in individual preparations. Three perforators were identified in 29 elevated flaps (48.33 %); two perforators, in 23 (38.33 %), and one perforator, in 7 (11.67 %). In one case (1.67 %), no perforators were identified. Blood vessels were present in various configurations, as shown in a bar graph in Fig. 4. An average number of perforators per flap were 2.33. Both, in men as well as in women, clinically relevant perforators (*Φ* > 0.5 mm) significantly more often occurred as A or B perforators (male *χ* ^2^ = 11.72, *p* = 0.0006; female *χ* ^2^ = 6.65, *p* = 0.009) (Fig. 5). Moreover, in both sexes, perforator A was most often identified as thick perforator. This observation was statistically significant (male *χ* ^2^ = 17.19, *p* < 0.000001; female *χ* ^2^ = 18.04, *p* < 0.000001). On the other hand, thin perforators (<0.5 mm) were in majority of cases located distally, as C perforators. Mean thickness of the fasciocutaneous flaps measured in the central part in the spine–patella segment was 3.65 cm in women and 1.17 cm in men (difference was statistically significant; *t* = −14.444, *p* < 0.00001).

**Table 1:** Number of perforators of different type and size observed in dissected thighs

		Perforators [*n* (%)]		
	Thick	Medium	Thin	Sum
Female				
Muscular	10	15	20	**45** (**32.1**)
Septal	13	7	4	**24** (**17.1**)
All	23	22	24	**69** (**49.2**)
Male				
Muscular	16	23	14	**53** (**37.9**)
Septal	11	4	3	**18** (**12.9**)
All	27	27	17	**71** (**50.8**)
Sum	**50** (**35.7**)	**49** (**35.0**)	**41** (**29.3**)	**140** (**100**)

*bold* entries at the bottom refer to the sum of all perforators in males and females (septal +muscular)

*bold* entries in the right column refer to the sum of "thick", "medium" and "thin" perforators

**Fig. 6:**
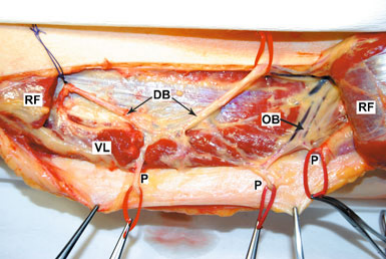
In this case, perforators originate from the descending and oblique branches. *OB* oblique branch, *DB* descending branch, *P* perforator, *VL* vastus lateralis, *RF* rectus femoris (cut)

In majority of cases, the flap was supplied from the descending branch. However, in men, the descending branch was the main blood vessel for 63 perforators, and seven perforators were supplied by the oblique branch. In women, the descending artery supplied 67 perforators, and the oblique branch, one perforator (Fig. 6).

## Discussion

Free flaps have been in use for more than three decades. In this time, survival of the flaps increased from 79 to 96 % (in majority of centers) [6]. Thus, in the current stage of development of microsurgery, the main emphasis is attached to functionality and appearance of both the donor and recipient sites [24–27]. Few donor sites allow obtaining so ample amounts of tissue. ALTF is thin and pliable and easy to prepare, and its harvest is associated with relatively little damage to the donor site [23].

The first description of ALTF suggested that the flap blood supply depends exclusively on septal vessels [1]. The finding of the presence of musculocutaneous perforators, with the lack of septal vessels, was considered a contraindication to the procedure. Koshima in his first reports remarked that ALTF could not have been applied in five out of 13 cases due to lack of septocutaneous vessels [11].

Preparation of muscular perforators became possible, safe, and more popularized later on, with progress in the studies on perforator flaps. It appeared that this type of vessels is most commonly encountered in the anterolateral flap. According to some authors, muscular perforators constitute 60 %, and septal, 40 % of all perforators [10]. Other reports point to even more significant predominance of musculocutaneous perforators—81.9 vs 18.1 % [7] and 87.1 vs 12.9 % [6]. Satish and Baliarsing put stress on coexisting, both types of perforators in 18 % of cases [28]. In our study, the percentage of musculocutaneous and septocutaneous perforators was 70 and 30 %, respectively.

The situations when no perforators are identified within the anterolateral surface of the thigh are rare. Lin and Yu in a series of 250 flaps demonstrated lack of perforators in 12 cases (4.8 %); in two of them, anteromedial flap was used, and in further ten cases, the flaps were harvested from the other limb. Four of them did not contain any perforating vessels (1.6 %) [16]. In the study conducted by Kimata, no perforators were found in 5.4 % of cases [21]. Some of the authors estimate that lack of perforators is observed in about 1 % of cases [19, 22]. This discrepancy may arise from the fact that perforators with a small diameter (<1 mm) were not always qualified (especially in early series) or ignored. In our study, cases in which no perforators were identified constituted 1.6 % (two flaps).

Song et al. observed that septal ALTF vessels are located in the proximal one third of the thigh [1]. Subsequent reports point to a more distal location of majority of perforators, approximately in the middle of the line joining ASIS with superolateral border of the patella. According to Malhorta, 65 % of vessels are located within 5 cm of this point [15]. However, Kimata in a series of 74 cases observed that many vessels are located in the middle of the thigh (point 0.5), but also a little more proximately, in the points 0.4 and 0.33 [21]. Yu et al. estimate that majority of vessels (45 %) are located in point 0.5, while the remaining are localized in points 0.4 (23 %) and 0.6 (31 %). However, the data concerned were not divided into genders [17]. In our study, the AP coefficient was 0.56 for women and 0.5 for men.

It is assumed that the skin on the ALTF is supplied from the LCFA descending branch. However, it may happen in some cases that the function is performed by another artery. Some authors, in their anatomical studies observed this “abnormal” blood supply. Kimata assessed that this anatomical variant occurs in 16 % of cases [21]; Shieh et al. reported even more common occurrence of 32 % [3]. In a prospective analysis of 89 anterolateral flaps, Wong et al. observed this so far unknown “nameless” branch in 34 % of cases and gave it the name of “oblique branch” [22]. Its significance in clinical practice is undeniable. The vastus lateralis muscle is supplied by the descending branch of the LCFA; usually, the skin and subcutaneous tissue from this region receive blood supply from this artery through the perforating vessels. Sometimes, however, it may happen that the muscle and skin have diverse sources of blood supply—the descending branch and oblique branch, respectively. This may explain a situation when in an elevated anterolateral thigh flap including the vastus lateralis muscle “en masse,” the muscle receives normal blood supply, while the skin becomes necrotic. This occurs because the oblique branch, which supplies the skin, is ligated during dissection of the muscle in its proximal part [29]. The presence of this alternative blood supply to the ALTF is advantageous; thanks to it, the whole descending branch can be spared, and thus, blood supply to the vastus lateralis muscle is maintained. According to Wei et al. and Mardini et al. [30, 31], almost every skin vessel diagnosed acoustically by means of hand-held Doppler may be prepared in retrograde manner and used as a base for a flap. In view of this idea, the descending branch does not have to constitute a vascular pedicle of the ALTF, and it may be replaced by any artery to which leads a skin vessel during retrograde preparation, e.g., the oblique branch. This causes that the ALTF is a kind of “free style flap” (Wei [6]). The branch occurs with various frequencies. In our study, this type of vascularization was identified in 13.3 % of cases, and taking into account a division into sexes, in men, the oblique branch occurred in 23.3 % of cases, and in women, only in 3.3 %.

## Conclusion

Our cadaveric study demonstrates some differences in ALTF in both sexes. In men, perforators are located closer to the anterior superior iliac spine in comparison to women. Also, the thickness of the flap is statistically higher in women than in men. In both sexes, there may exist an alternative blood supply—oblique branch, but it is statistically more common in men. In both sexes, clinically significant perforators (*Φ* > 0.5 mm), in majority of cases, occur in A and B positions.

## References

[1] Song YG, Chen GZ, Song YL (1984). The free thigh flap: a new free flap concept based on the septocutaneous artery. Br J Plast Surg.

[2] Lutz BS, Wei F-C (2005). Microsurgical workhorse flaps in head and neck reconstruction. Clin Plast Surg.

[3] Shieh SJ, Chiu HY, Yu JC, Pan SC, Tsai ST, Shen CL (2000). Free anterolateral thigh flap for reconstruction of head and neck defects following cancer ablation. Plast Reconstr Surg.

[4] Demirkan F, Chen HC, Wei F-C, Chen HH, Jung SG, Hau SP (2000). The versatile anterolateral thigh flap: a musculocutaneous flap in disguise in head and neck reconstruction. Br J Plast Surg.

[5] Koshima I (2000). Free anterolateral thigh flap for reconstruction of head and neck defects following cancer ablation. Plast Reconstr Surg.

[6] Wei F-C, Jain V, Celik N, Chen HC, Chuang DC, Lin CH (2002). Have we found an ideal soft-tissue flap? An experience with 672 anterolateral thigh flaps. Plast Reconstr Surg.

[7] Kimata Y, Uchiyama K, Ebihara S, Yoshizumi T, Asai M, Saikawa M (1997). Versatility of the free anterolateral thigh flap for reconstruction of head and neck defects. Arch Otolaryngol Head Neck Surg.

[8] Luo S, Raffoul W, Luo J, Luo L, Gao J, Chen L (1999). Anterolateral thigh flap: a review of 168 cases. Microsurgery.

[9] Mardini S, Lin LC, Moran SL, Saldago CJ, Wei F-C, Wei F-C, Mardini S (2009). Anterolateral thigh flap. Flaps and reconstructive surgery.

[10] Xu DC, Zhong SZ, Kong JM, Wang GY, Liu MZ, Luo LS (1988). Applied anatomy of the anterolateral femoral flap. Plast Reconstr Surg.

[11] Koshima I, Fukuda H, Utunomiya R, Soeda S (1989). The anterolateral thigh flap; variations in its vascular pedicle. Br J Plast Surg.

[12] Zhou G, Qiao Q, Chen GY, Ling YC, Swift R (1991). Clinical experience and surgical anatomy of 32 free anterolateral thigh flap transplantations. Br J Plast Surg.

[13] Pribaz JJ, Orgill DP, Epstein MD, Sampson CE, Hergrueter CA (1995). Anterolateral thigh free flap. Ann Plast Surg.

[14] Mäkitie AA, Beasley NJ, Neligan PC, Lipa J, Gullane PJ, Gilbert RW (2003). Head and neck reconstruction with anterolateral thigh flap. Otolaryngol Head Neck Surg.

[15] Malhotra K, Lian TS, Chakradeo V (2008). Vascular anatomy of anterolateral thigh flap. Laryngoscope.

[16] Lin SJ, Rabie A, Yu P (2010). Designing the anterolateral thigh flap without preoperative Doppler or imaging. J Reconstr Microsurg.

[17] Yu P (2004). Characteristics of the anterolateral thigh flap in a Western population and its application in head and neck reconstruction. Head Neck.

[18] Kawai K, Imanishi N, Nakajima H, Aiso S, Kakibuchi M, Hosokawa K (2004). Vascular anatomy of the anterolateral thigh flap. Plast Reconstr Surg.

[19] Rozen WM, Ashton MW, Pan WR, Kiil BJ, McClure VK, Grinsell D (2009). Anatomical variations in the harvest of anterolateral thigh flap perforators: a cadaveric and clinical study. Microsurgery.

[20] Chen Z, Zhang C, Lao J, Xing JJ, Zhu MX, Wu Y (2007). An anterolateral thigh flap based on the superior cutaneous perforator artery: an anatomic study and case reports. Microsurgery.

[21] Kimata Y, Uchiyama K, Ebihara S, Nakatsuka T, Harii K (1998). Anatomic variations and technical problems of the anterolateral thigh flap: a report of 74 cases. Plast Reconstr Surg.

[22] Wong CH, Wei F-C, Fu B, Chen YA, Lin JY (2009). Alternative vascular pedicle of the anterolateral thigh flap: the oblique branch of the lateral circumflex femoral artery. Plast Reconstr Surg.

[23] Chana JS, Wei F-C (2004). A review of the advantages of the anterolateral thigh flap in head and neck reconstruction. Br J Plast Surg.

[24] Blackwell KE, Brown MT, Gonzalez D (1997). Overcoming the learning curve in microvascular head and neck reconstruction. Arch Otolaryngol Head Neck Surg.

[25] Cordeiro PG, Hidalgo DA (1995). Conceptual considerations in mandibular reconstruction. Clin Plast Surg.

[26] Santamaria E, Wei FC, Chen HC (1998). Fibula osteoseptocutaneous flap for reconstruction of osteoradionecrosis of the mandible. Plast Reconstr Surg.

[27] Hidalgo DA (1991). Aesthetic improvements in free-flap mandible reconstruction. Plast Reconstr Surg.

[28] Satish C, Baliarsing A (2012). Variations in perforators of anterolateral thigh flap. Eur J Plast Surg.

[29] Wong CH, Kao HK, Fu B, Lin JY (2009). A cautionary point in the harvest of the anterolateral thigh myocutaneous flap. Ann Plast Surg.

[30] Wei F-C, Mardini S (2004). Free-style free flaps. Plast Reconstr Surg.

[31] Mardini S, Tsai FC, Wei F-C (2003). The thigh as a model for free style free flaps. Clin Plast Surg.

